# Suturing Dermatotraction Techniques in Closing Fasciotomy Wounds: A Systematic Review

**DOI:** 10.7759/cureus.37550

**Published:** 2023-04-13

**Authors:** Otomi O Obuh, Ena-Jane O Esomu, Roseline O Sydney

**Affiliations:** 1 Plastic and Reconstructive Surgery, Surgery Interest Group of Africa, Lagos, NGA; 2 Surgery, Imperial College Healthcare NHS Trust, London, GBR; 3 Surgery, University of Benin, Benin City, NGA; 4 General Practice, University of Ibadan, Ibadan, NGA

**Keywords:** wound closure, shoelace, dermatotraction, compartment syndrome, fasciotomy

## Abstract

A surgical patient post-fasciotomy presents a challenge to restore the cover of the muscle groups, and the use of the suturing dermatotraction techniques presents a cheap and easy means of native cover. This systematic review of case series and case-control study explored the trend of this technique, including duration of delayed primary wound closure, complications, and failure rates. A literature review following Preferred Reporting Items for Systematic Reviews and Meta-Analyses (PRISMA) guidelines was conducted on Medline, Embase, and Cumulative Index of Nursing and Allied Health Literature (CINAHL), yielding a combined total of 820 articles between 1946 and June 18, 2022. Human studies with suturing dermatotraction techniques were included. Sixteen (16) studies reviewed met the criteria. The basic anatomy of the dermatotraction technique involves an anchor point on the skin, a material for traction, and a suture pattern. The shoelace technique was the predominant suture pattern, with staples as skin anchor material/method and silastic vessel loops as traction sling used by 11 studies. Modifications of this method included the use of intradermal Prolene sutures and pediatric catheters. The shortest duration for skin apposition was two days, and the longest was 113 days. Complications were comparable to that of surgical wounds and thus may not be attributable to the technique itself. Studies reviewed showed that superficial and early complications were more likely than deep or delayed complications. Negative pressure wound therapy (NPWT) and skin graft salvaged a few failed closures in two studies. There are varying practices of tightening rates with reports ranging from daily to every 72 hours. The rate of tightening and disease burden may account for the wide range of reported delayed primary closure. Most of the studies reviewed closed fasciotomy wounds with this technique within an average of <10 days. It is relatively cheaper, carries a low morbidity burden, and has multiple reported success in the closure of fasciotomy wounds in this review and thus should have an increased adoption as a first approach in managing fasciotomy wounds, especially in low-income countries.

## Introduction and background

The surgeon, when faced with an acute limb due to compartment syndrome, faces two critical challenges: the emergent need to decompress the compartment syndrome by performing a fasciotomy and the inevitable need to promptly return superficial closure to the muscle groups as close as possible to their natural state or else risk the antecedent complications of loss of skin coverage and its highly revered role as the first line of defense against infections and maintenance of homeostasis of the underlying tissue. A surgical patient post-fasciotomy thus presents a challenge to restore the cover of the muscle groups [1,2].

It is much accepted that wounds ought to be covered as soon as reasonable to prevent complications from arising [3,4]. There are however various methods in the surgeon’s armamentarium to deploy to close the wound. The use of the suturing techniques presents a cheap and easy means of closing fasciotomy wounds; it is also a basic surgical skill to develop [5-7].

The use of the suturing dermatotraction techniques presents a cheap and easy means of native cover [8]. This systematic review of case series and case-control study explored the trend of this technique, particularly the duration of delayed primary wound closure, complications, and failure rates.

## Review

Methods

The inclusion and exclusion criteria were tailored to answer the questions of this review, which centered on finding out the trend and practice of the use of dermatotraction techniques using sutures in the existing literature and identifying successes associated with this practice and complications. To this end, all studies included were human studies in patients who had undergone a fasciotomy procedure for the management of an acute limb compartment syndrome. There was no exclusion based on the limb affected. Furthermore, these studies had to have had a dermatotraction technique as one of their means of closure of fasciotomy wounds. Textbooks, letters to editors, commentaries, review studies, case series involving <2 patients, and single case reports were excluded; however, their bibliographies were consulted to ensure all eligible articles were captured. All articles reviewed and retrieved were in English.

**Figure 1:**
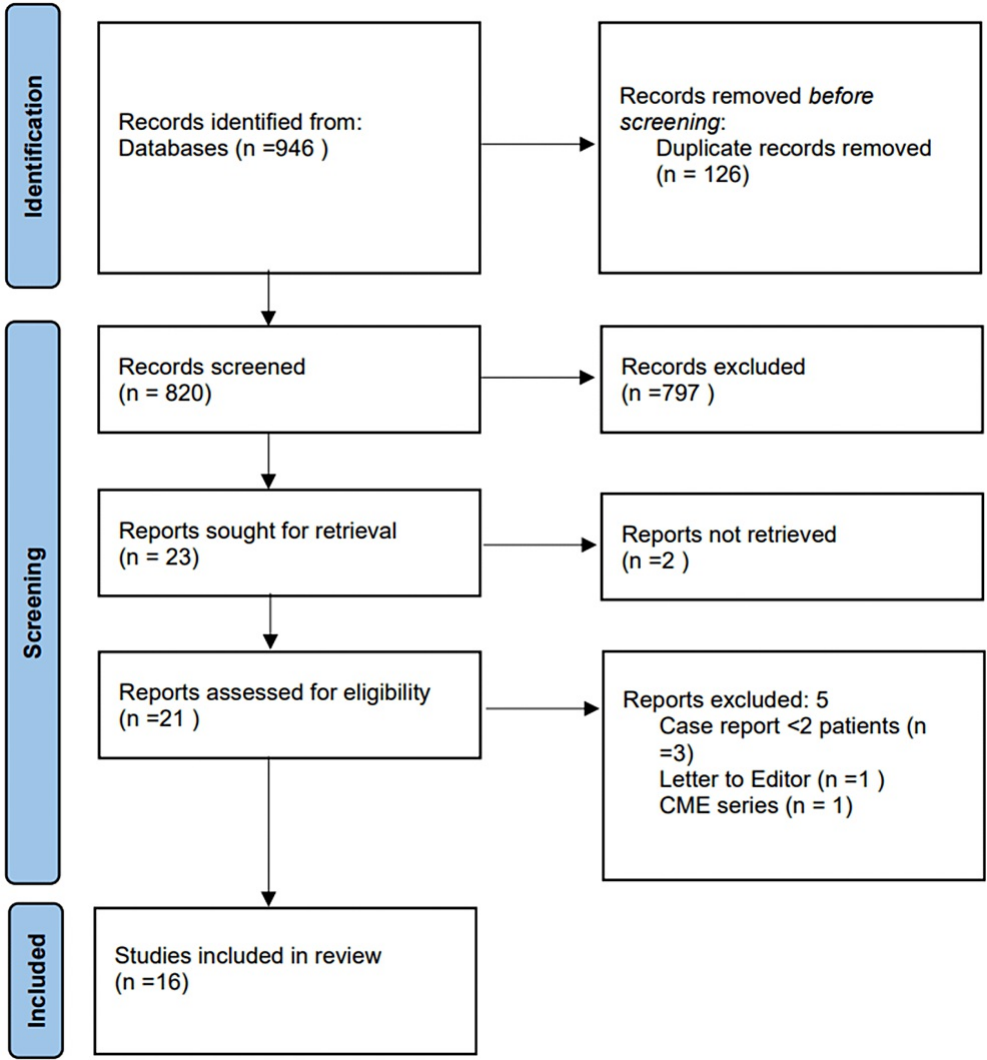
PRISMA flowchart PRISMA: Preferred Reporting Items for Systematic Reviews and Meta-Analyses, CME: continuing medical education

A thorough literature search was conducted between 1946 and June 18, 2022, using Preferred Reporting Items for Systematic Reviews and Meta-Analyses (PRISMA) principles. The search strategy was based on MEDLINE using the Medical Subject Headings (MeSH) terms wound closure techniques, suture techniques, sutures, and fasciotomy; these were then adapted to other databases of Embase and Cumulative Index of Nursing and Allied Health Literature (CINAHL) via OVID (Appendices). Titles and abstracts were screened by two contributors in a blinded approach using the predetermined inclusion and exclusion criteria; studies meeting these criteria were further reviewed in full text. In cases of a conflict of literature selection, a third contributor re-reviewed and made a final decision. The search yielded a total of 820 articles after removing duplicated articles, and 16 articles met the criteria for full-text review (Figure 1).

Results

In keeping with the aims of the project, we identified the suturing dermatotraction technique used and the average number of days to achieve closure, including the range, as well as additional methods used to close wounds, complications, and if there was complete fasciotomy closure in most cases, i.e., >50%. Studies were published between 1993 and 2021. There were 14 case series, one case-control study, and one RCT (Table 1).

**Table 1:** Results of the literature search NWPT: negative pressure wound therapy

Author	Year	Number of patients	Study type	Suture/traction material used	Suturing dermatotraction technique	Average duration to achieve closure	Range to achieve closure (days)	Complete wound closure achieved in most patients	Add-on method ab initio	Additional method needed to close wound(s)	Record of failed fasciotomy wound closure	Recorded complications	Rate of tightening
Zorrilla et al. [9]	2005	20	Retrospective case series	Vessel loops	Shoelace with surgical staples at 1.5-2 cm intervals	8.8 days	6-19 days	Yes	No	No	No	5% retractile scar causing limitation to the passive extension of the joint proximal to the scar	48 hours
Kakagia [6]	2014	25	Prospective case series	Vessel loops	Shoelace with surgical staples	15.1 days	11-30 days	Yes	No	No	No	16% infection with *Staphylococcus epidermidis*, *Pseudomonas*, and *Acinetobacter baumannii*	Daily
Asgari et al. [10]	2000	37	Case series	Vessel loops	Shoelace with surgical staples at 1 cm intervals and 0.3-0.5 cm from the wound edge	12 days	Equal/lesser than 3 weeks	Yes	No	Use of surgical staples or 3.0/4.0 nylon suture after the vessel loop had been removed	No	No	Daily
Eid et al. [11]	2012	17	Case series	Pediatric urinary catheter and surgical skin staples	Shoelace with staples	3.8 weeks	No data	Yes	No	Dynamization and bone grafting in eight patients either to assist in the healing of the fracture or remove the implant (intramedullary nail)	No	No	48-72 hours
Chiverton et al. [12]	2000	6	Case series	4/0 nylon sutures and 2/0 or 6/0 Prolene sutures	Interrupted vertical mattress nylon technique and subcuticular Prolene technique	Not clear	1-3 days	Yes	No	No	No	No	Not specific
Johnson et al. [13]	2018	5	Randomized controlled trial	Not clear	Shoelace with surgical staples	7.6 days	No data	Yes	No	No	No	No	48 hours
Suomalainen et al. [14]	2021	47	Retrospective case series	Vessel loops	Shoelace with surgical staples at 0.5 cm from the wound edge	5.9 days	2-19 days	Yes in 36 patients	No	No	Yes in 11 patients, free flap used as an intervention	Infection	Daily
Arumugam et al. [15]	2020	8	Prospective case-control study	Vessel loops	Shoelace with surgical staples	7 days	6-10 days	Yes	No	No	No	No	48-72 hours
Ozyurtlu et al. [16]	2014	5	Case series	Barbed suture	Intradermal, horizontal mattress	8.6 days	6-14 days	Yes	No	No	No	One case of skin necrosis	48-72 hours
Fowler et al. [17]	2012	49	Retrospective case series	Vessel loops	Shoelace with surgical staples	19.2 days in admission (days stated as the duration of admission)	3-113 days in admission (days stated as the duration of admission)	Yes	No	No	Yes in nine patients	6.67% infection (three patients)	Not specific
Dodenhotf et al. [18]	1997	20	Case series	Vessel loops	Shoelace with surgical staples	6 days	4-10 days	Yes	No	No	Yes in one patient, free flap used as an intervention	None	Not specific
Harris [19]	1993	5	Case series	Vessel loops	Shoelace with surgical staples at intervals of 1.5- 2 cm	9 days	7-11 days	Yes	No	No	No	No	Not specific
Zenke et al [20]	2014	5	Case series	Vessel loops	Shoelace with surgical staples	16.2 days	9-27 days	Yes	Yes (NPWT)	No	Yes in one patient, skin graft used as an intervention	Yes, partial wound necrosis	Not specific
Eceviz et al. [21]	2020	7	Case series	Vessel loops	Shoelace with surgical staples	11.8 days	5-30 days	Yes	No	No	Yes, NPWT used as an intervention	Wound infection	48 hours
Mittal et al. [22]	2018	25	Comparative case series	Ethilon suture	Shoelace with corrugated drains	10 days	Not stated	Yes	No	No	Yes in one patient	Wound infection	Not specific
Janzing et al. [23]	2001	10	Case series	Vessel loop (5), monofilament (5)	Shoelace with staples, prepositioned sutures	9 days	Not stated	Yes	No	No	No	Delayed compartment syndrome (one patient)	Not specific

Discussion

Suturing Style

**Figure 2:**
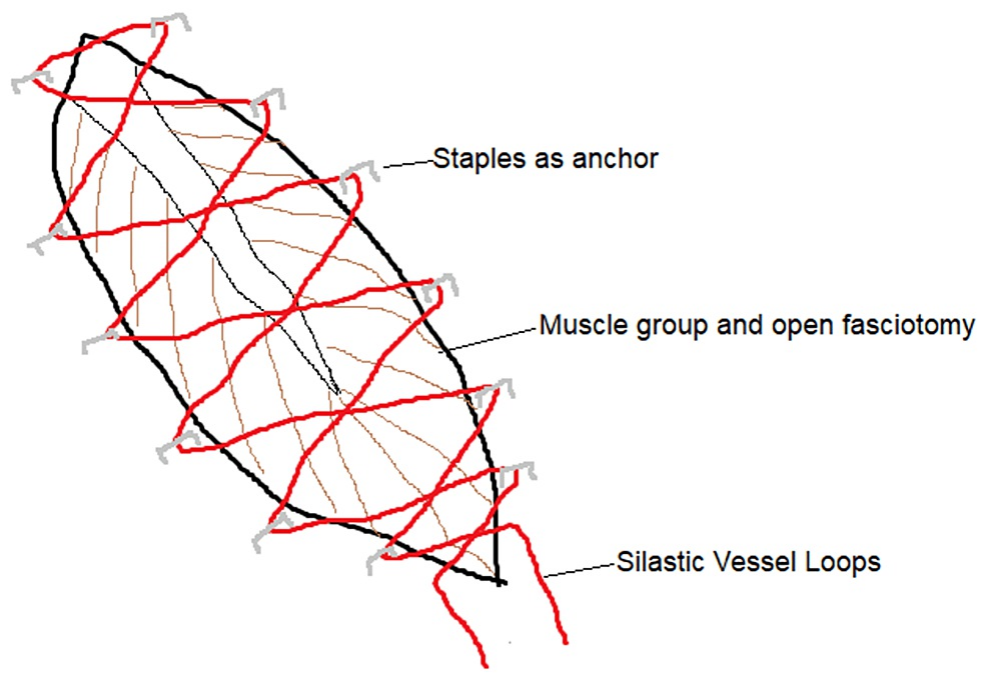
Anatomy of the shoelace technique

The basic anatomy of the dermatotraction suture technique involves an anchor point on the skin/through the skin, a material with properties that enable traction, and a knotting pattern that enables uniform application of traction forces through the pulleys (Figure 2) [24,25]. There are various modifications to this architecture, and various authors have come up with their own adaptations in case reports [26]. The intervals of staples were between 1 and 2 cm in some studies, and the distance from the staples to the wound edge was reported as 0.3-0.5 cm in other studies.

The shoelace technique as described was the predominant knotting pattern, with staples as the predominant skin anchor material/method and silastic vessel loops as the predominant traction sling used by 11 studies. Varying modifications of this method included the use of a pediatric urinary catheter as the sling for traction and surgical staples, nylon sutures, Prolene sutures, and barbed suture, with a subcuticular, interrupted vertical and horizontal mattress technique as combinations of both anchor and stretching material, and the use of shoelace with corrugated drains using Ethilon sutures for dermatotraction, all in single cases. In one case, an add-on technique using surgical staples after the removal of the vessel loop was employed; in another, bone grafting was used as it suited the cohort of patients being treated; and in one study, a combination with NWPT ab initio was used.

The rate of tightening was reported as daily in three studies, every 48 hours in three studies, and 48-72 hours in three studies. Other studies used terms such as gradual tightening, which would appear subjective to the operator. A study used the capillary refill time of wound edges as an adjunct to tell how much tightening was allowable with each session [21]. Some tightening was recorded as done under a sedative, general anesthesia, and no anesthesia/analgesia, probably reflecting patient factors in influencing the choice [19], and wound inspections were carried out in most studies in various clinical settings, including theater and by the bedside.

Varying modifications to the use of dermatotraction suture techniques have also been employed in other body wounds with good results [27,28], and there is an emerging technology that does not require manual tightening [29]. It could not be clearly concluded due to limited data if add-on methods such as NWPT or varied modifications offered any advantage in the management of lower limb fasciotomies.

Time to Achieve Primary Closure

For all the reports included in the study, the average time to complete the closure of the fasciotomy wound using the dermatotraction suture technique was the shortest at 5.9 days and the longest at 3.8 weeks. Nine studies had an average duration of closure of within 10 days, four studies within >10 days, and one study within <17 days. In extremis, there was a single study with an average duration of 3.8 weeks. The shortest duration for complete primary closure was two days and the longest was 113 days; the reason for this prolonged duration and discrepancies were not immediately clear. Ten out of 16 studies reported a complete closure of fasciotomy wounds, and all studies had closure in most of the patients included. Four cases had one patient failing a full cover and needing skin grafting and NWPT to treat failed closure. In the study with the largest data set, only 23% of patients had a failed closure. The success of this method is also re-echoed in other studies [8]. It was not clear from many of the studies the extent and size of the wounds and where this was stated if this contributed to failure rates, wound closure time, or if patient factors might have contributed. A study however found no statistically significant correlation between wound closure time and wound length [6], in contrast to another study that found a correlation to wound closure time [23].

Complications

Most studies showed that the shoelace technique would largely have fewer or no complications in comparison to other techniques of fasciotomy wound closure. Superficial and early complications were more likely than deep or delayed complications.

The most recorded complication in the reviewed series was an infection, with one study adding specific causative organisms in 16% as *Staphylococcus epidermidis*, *Pseudomonas*, and *Acinetobacter baumannii*. Postoperative retractile scaring was noted in one series, and partial skin necrosis was another common complication reported in two out of 16 studies. These recorded complications are known to be associated with delayed primary wound closure [30,31].

Fasciotomy wounds in general are not clean wounds. The reported infection rates are as high as 30% [32] and as low as 16.7% [33], and this falls within the range stated for surgical site infections (SSIs) in the general series of 2%-20% [34]. However, as with all wounds, the factors determining infection and these complications would be expected to include the extent of morbidity and host factors.

## Conclusions

The dermatotraction suture techniques with their modifications present a cheap, readily available technique for closing fasciotomies. This review was largely limited by the heterogeneous nature of the articles and their objectives, techniques were not clearly defined in most studies, and it was therefore difficult to extrapolate what most authors adopted. There is a need for more detailed studies that investigate this largely successful closure technique. We would recommend future studies incorporating details such as the disease burden of the wound and a standardized method of application of this technique. Case-control studies or randomized controlled trials might be best suited to answer this; however, as is known in the world of surgical innovation, standardization of techniques can be challenging, and refined patient selection and standardized training would be reasonable first steps.

Most fasciotomy wounds closed with suturing techniques can expect to close within 10 days, with minimal complications. There are varying practices of traction rates, and this may account for the wide range of delayed primary closure. This review has highlighted the simple anatomy of this technique, which in all ramifications is also affordable and accessible, especially in the context of low-resource countries, where we have had experience practicing. The success rates are reasonable, and complications are not unexpected in keeping with wound healing. It is relatively cheaper, carries a low morbidity burden, and has multiple reported success in the closure of fasciotomy wounds in this review and thus should have an increased adoption as a first approach in managing fasciotomy wounds, especially in low-income countries. We hope this review will spur increased confident uptake of this technique in low-resource countries and, given their proven efficiency, become a ready tool in the armamentarium of trauma and vascular surgeons in managing fasciotomy wounds.

## Appendices

Search strategy

Ovid MEDLINE(R) ALL <1946 to June 13, 2022>

1 vessel loop*.mp. [mp=title, abstract, original title, name of substance word, subject heading word, floating sub-heading word, keyword heading word, organism supplementary concept word, protocol supplementary concept word, rare disease supplementary concept word, unique identifier, synonyms] 193

2 shoe lac*.mp. [mp=title, abstract, original title, name of substance word, subject heading word, floating sub-heading word, keyword heading word, organism supplementary concept word, protocol supplementary concept word, rare disease supplementary concept word, unique identifier, synonyms] 22

3 shoelac*.mp. [mp=title, abstract, original title, name of substance word, subject heading word, floating sub-heading word, keyword heading word, organism supplementary concept word, protocol supplementary concept word, rare disease supplementary concept word, unique identifier, synonyms] 107

4 (dermal adj2 apposit*).mp. [mp=title, abstract, original title, name of substance word, subject heading word, floating sub-heading word, keyword heading word, organism supplementary concept word, protocol supplementary concept word, rare disease supplementary concept word, unique identifier, synonyms] 9

5 gradual primary clos*.mp. [mp=title, abstract, original title, name of substance word, subject heading word, floating sub-heading word, keyword heading word, organism supplementary concept word, protocol supplementary concept word, rare disease supplementary concept word, unique identifier, synonyms] 3

6 (delayed adj2 "primary closure").mp. [mp=title, abstract, original title, name of substance word, subject heading word, floating sub-heading word, keyword heading word, organism supplementary concept word, protocol supplementary concept word, rare disease supplementary concept word, unique identifier, synonyms] 361

7 Dynamic wound clos*.mp. [mp=title, abstract, original title, name of substance word, subject heading word, floating sub-heading word, keyword heading word, organism supplementary concept word, protocol supplementary concept word, rare disease supplementary concept word, unique identifier, synonyms] 8

8 (subcuticular adj3 sutur*).mp. [mp=title, abstract, original title, name of substance word, subject heading word, floating sub-heading word, keyword heading word, organism supplementary concept word, protocol supplementary concept word, rare disease supplementary concept word, unique identifier, synonyms] 384

9 (intracutaneous adj3 sutur*).mp. [mp=title, abstract, original title, name of substance word, subject heading word, floating sub-heading word, keyword heading word, organism supplementary concept word, protocol supplementary concept word, rare disease supplementary concept word, unique identifier, synonyms] 66

10 dermatotract*.mp. [mp=title, abstract, original title, name of substance word, subject heading word, floating sub-heading word, keyword heading word, organism supplementary concept word, protocol supplementary concept word, rare disease supplementary concept word, unique identifier, synonyms] 19

11 Sure clos*.mp. [mp=title, abstract, original title, name of substance word, subject heading word, floating sub-heading word, keyword heading word, organism supplementary concept word, protocol supplementary concept word, rare disease supplementary concept word, unique identifier, synonyms] 13

12 (skin adj2 approxim*).mp. [mp=title, abstract, original title, name of substance word, subject heading word, floating sub-heading word, keyword heading word, organism supplementary concept word, protocol supplementary concept word, rare disease supplementary concept word, unique identifier, synonyms] 447

13 (sutur* adj2 approxim*).mp. [mp=title, abstract, original title, name of substance word, subject heading word, floating sub-heading word, keyword heading word, organism supplementary concept word, protocol supplementary concept word, rare disease supplementary concept word, unique identifier, synonyms] 186

14 Wound Closure Techniques/ 1802

15 Suture Techniques/ 44529

16 Sutures/ 18752

17 1 or 2 or 3 or 4 or 5 or 6 or 7 or 8 or 9 or 10 or 11 or 12 or 13 or 14 or 15 or 16 59920

18 Fasciectom*.mp. [mp=title, abstract, original title, name of substance word, subject heading word, floating sub-heading word, keyword heading word, organism supplementary concept word, protocol supplementary concept word, rare disease supplementary concept word, unique identifier, synonyms] 506

19 Fasciotom*.mp. [mp=title, abstract, original title, name of substance word, subject heading word, floating sub-heading word, keyword heading word, organism supplementary concept word, protocol supplementary concept word, rare disease supplementary concept word, unique identifier, synonyms] 6166

20 Fasciotomy/ 3994

21 18 or 19 or 20 6365

22 17 and 21 560

https://ovidsp.ovid.com/ovidweb.cgi?T=JS&NEWS=N&PAGE=main&SHAREDSEARCHID=45mN4kAJ3Nm4ZPX0Ae88IuM79P4hjsXces3f40j5bknehO2KJ5ZKSe0niZtVUQZhm

Embase <1974 to 2022 June 13>

1 vessel loop*.mp. [mp=title, abstract, heading word, drug trade name, original title, device manufacturer, drug manufacturer, device trade name, keyword heading word, floating subheading word, candidate term word] 380

2 shoe lac*.mp. [mp=title, abstract, heading word, drug trade name, original title, device manufacturer, drug manufacturer, device trade name, keyword heading word, floating subheading word, candidate term word] 35

3 shoelac*.mp. [mp=title, abstract, heading word, drug trade name, original title, device manufacturer, drug manufacturer, device trade name, keyword heading word, floating subheading word, candidate term word] 149

4 (dermal adj2 apposit*).mp. [mp=title, abstract, heading word, drug trade name, original title, device manufacturer, drug manufacturer, device trade name, keyword heading word, floating subheading word, candidate term word] 10

5 gradual primary clos*.mp. [mp=title, abstract, heading word, drug trade name, original title, device manufacturer, drug manufacturer, device trade name, keyword heading word, floating subheading word, candidate term word] 2

6 (delayed adj2 "primary closure").mp. [mp=title, abstract, heading word, drug trade name, original title, device manufacturer, drug manufacturer, device trade name, keyword heading word, floating subheading word, candidate term word] 432

7 Dynamic wound clos*.mp. [mp=title, abstract, heading word, drug trade name, original title, device manufacturer, drug manufacturer, device trade name, keyword heading word, floating subheading word, candidate term word] 10

8 (subcuticular adj3 sutur*).mp. [mp=title, abstract, heading word, drug trade name, original title, device manufacturer, drug manufacturer, device trade name, keyword heading word, floating subheading word, candidate term word] 493

9 (intracutaneous adj3 sutur*).mp. [mp=title, abstract, heading word, drug trade name, original title, device manufacturer, drug manufacturer, device trade name, keyword heading word, floating subheading word, candidate term word] 106

10 dermatotract*.mp. [mp=title, abstract, heading word, drug trade name, original title, device manufacturer, drug manufacturer, device trade name, keyword heading word, floating subheading word, candidate term word] 20

11 Sure clos*.mp. [mp=title, abstract, heading word, drug trade name, original title, device manufacturer, drug manufacturer, device trade name, keyword heading word, floating subheading word, candidate term word] 23

12 skin approxim*.mp. [mp=title, abstract, heading word, drug trade name, original title, device manufacturer, drug manufacturer, device trade name, keyword heading word, floating subheading word, candidate term word] 119

13 sutur* approxim*.mp. [mp=title, abstract, heading word, drug trade name, original title, device manufacturer, drug manufacturer, device trade name, keyword heading word, floating subheading word, candidate term word] 70

14 wound closure/ 20305

15 suture/ 40426

16 suture technique/ 6200

17 1 or 2 or 3 or 4 or 5 or 6 or 7 or 8 or 9 or 10 or 11 or 12 or 13 or 14 or 15 or 16 65340

18 Fasciectom*.mp. [mp=title, abstract, heading word, drug trade name, original title, device manufacturer, drug manufacturer, device trade name, keyword heading word, floating subheading word, candidate term word] 599

19 Fasciotom*.mp. [mp=title, abstract, heading word, drug trade name, original title, device manufacturer, drug manufacturer, device trade name, keyword heading word, floating subheading word, candidate term word] 6899

20 fasciotomy/ 5737

21 18 or 19 or 20 7280

22 17 and 21 328

https://ovidsp.ovid.com/ovidweb.cgi?T=JS&NEWS=N&PAGE=main&SHAREDSEARCHID=1JxCe2MF46e04dgqlc4wFU5aEPSj6kWpR5NMMWnh3kdF8QU68qshmrRcrheRnlMx7

Developing the search strategy

Concept 1: Shoelace Technique/Gradual Dermal Apposition Using Sutures

MESH:

Wound Closure Techniques/ 1802

Suture Techniques/ 44529

Sutures/ 18752

Keywords identified:

vessel loop shoe lace technique

dermal apposition

technique for gradual primary closure of fasciotomy wound

sub cuticular suture

Ty -Raps

Sure closure

Dynamic wound closure

STAR

Silver Bullet Wound Closure Device

vessel loops

dermatotraction

pre-positioned intracutaneous suture

Dermatotraction

silicon sheet

skin stretching

Dermotaxis

subcuticular prolene suture

Suture Techniques

fasciotomy closure

dynamic dermatotraction

gradual suture approximation

delayed primary closure

Dynamic wound closure

retention sutures

delayed primary closure (DPC)

vessel loops

the skin approximation system

prepositioned intracutaneous suture

Luggage tag tie

Concept 2: Fasciotomy Wound

MESH: Fasciotomy

-Fasciectomy (Probable alternative older term in literature)

## References

[1] Nguyen AV, Soulika AM (2019). The dynamics of the skin's immune system. Int J Mol Sci.

[2] Cone J, Inaba K (2017). Lower extremity compartment syndrome. Trauma Surg Acute Care Open.

[3] Jaman J, Martić K, Rasic N, Markulin H, Haberle S (2021). Is the use of specific time cut-off or "golden period" for primary closure of acute traumatic wounds evidence based? A systematic review. Croat Med J.

[4] Wood T, Sameem M, Avram R, Bhandari M, Petrisor B (2012). A systematic review of early versus delayed wound closure in patients with open fractures requiring flap coverage. J Trauma Acute Care Surg.

[5] Igoumenou VG, Kokkalis ZT, Mavrogenis AF (2019). Fasciotomy wound management. Compartment syndrome: a guide to diagnosis and management.

[6] Kakagia D (2015). How to close a limb fasciotomy wound: an overview of current techniques. Int J Low Extrem Wounds.

[7] Alkhalifah MK, Almutairi FS (2019). Optimising wound closure following a fasciotomy: a narrative review. Sultan Qaboos Univ Med J.

[8] Jauregui JJ, Yarmis SJ, Tsai J, Onuoha KO, Illical E, Paulino CB (2017). Fasciotomy closure techniques. J Orthop Surg (Hong Kong).

[9] Zorrilla P, Marín A, Gómez LA, Salido JA (2005). Shoelace technique for gradual closure of fasciotomy wounds. J Trauma.

[10] Asgari MM, Spinelli HM (2000). The vessel loop shoelace technique for closure of fasciotomy wounds. Ann Plast Surg.

[11] Eid A, Elsoufy M (2012). Shoelace wound closure for the management of fracture-related fasciotomy wounds. ISRN Orthop.

[12] Chiverton N, Redden JF (2000). A new technique for delayed primary closure of fasciotomy wounds. Injury.

[13] Johnson LS, Chaar M, Ball CG (2018). Management of extremity fasciotomy sites prospective randomized evaluation of two techniques. Am J Surg.

[14] Suomalainen P, Pakarinen TK, Pajamäki I, Laitinen MK, Laine HJ, Repo JP, Mattila VM (2021). Does the shoe-lace technique aid direct closure of fasciotomy wounds after acute compartment syndrome of the lower leg? A retrospective case-control study. Scand J Surg.

[15] Arumugam PK, Muthukumar V, Bamal R (2021). Utility of shoelace technique in closure of fasciotomy wounds in electric burns. J Burn Care Res.

[16] Ozyurtlu M, Altınkaya S, Baltu Y, Ozgenel GY (2014). A new, simple technique for gradual primary closure of fasciotomy wounds. Ulus Travma Acil Cerrahi Derg.

[17] Fowler JR, Kleiner MT, Das R, Gaughan JP, Rehman S (2012). Assisted closure of fasciotomy wounds: a descriptive series and caution in patients with vascular injury. Bone Joint Res.

[18] Dodenhoff RM, Howell GE (1997). The shoelace technique for wound closure in open fractures: report of early experience. Injury.

[19] Harris I (1993). Gradual closure of fasciotomy wounds using a vessel loop shoelace. Injury.

[20] Zenke Y, Inokuchi K, Okada H, Ooae K, Matsui K, Sakai A (2014). Useful technique using negative pressure wound therapy on postoperative lower leg open wounds with compartment syndrome. Inj Extra.

[21] Eceviz E, Çevik HB (2020). Shoelace technique plus negative-pressure wound therapy closure in fasciotomy wounds. Adv Skin Wound Care.

[22] Mittal N, Bohat R, Virk JS, Mittal P (2018). Dermotaxis v/s loop suture technique for closure of fasciotomy wounds: a study of 50 cases. Strategies Trauma Limb Reconstr.

[23] Janzing HM, Broos PL (2001). Dermatotraction: an effective technique for the closure of fasciotomy wounds: a preliminary report of fifteen patients. J Orthop Trauma.

[24] Berman SS, Schilling JD, McIntyre KE, Hunter GC, Bernhard VM (1994). Shoelace technique for delayed primary closure of fasciotomies. Am J Surg.

[25] Cohn BT, Shall J, Berkowitz M (1986). Forearm fasciotomy for acute compartment syndrome: a new technique for delayed primary closure. Orthopedics.

[26] Galois L, Pauchot J, Pfeffer F, Kermarrec I, Traversari R, Mainard D, Delagoutte JP (2002). Modified shoelace technique for delayed primary closure of the thigh after acute compartment syndrome. Acta Orthop Belg.

[27] Moran SG, Windham ST, Cross JM, Melton SM, Rue LW 3rd (2003). Vacuum-assisted complex wound closure with elastic vessel loop augmentation: a novel technique. J Wound Care.

[28] Govaert GA, van Helden S (2010). Ty-raps in trauma: a novel closing technique of extremity fasciotomy wounds. J Trauma.

[29] MacKay BJ, Dardano AN, Klapper AM, Parekh SG, Soliman MQ, Valerio IL (2020). Multidisciplinary application of an external tissue expander device to improve patient outcomes: a critical review. Adv Wound Care (New Rochelle).

[30] Bhangu A, Singh P, Lundy J, Bowley DM (2013). Systemic review and meta-analysis of randomized clinical trials comparing primary vs delayed primary skin closure in contaminated and dirty abdominal incisions. JAMA Surg.

[31] Siribumrungwong B, Noorit P, Wilasrusmee C, Thakkinstian A (2014). A systematic review and meta-analysis of randomised controlled trials of delayed primary wound closure in contaminated abdominal wounds. World J Emerg Surg.

[32] Merchan N, Ingalls B, Garcia J, Wixted J, Rozental TD, Harper CM, Dowlatshahi AS (2022). Factors associated with surgical site infections after fasciotomy in patients with compartment syndrome. J Am Acad Orthop Surg Glob Res Rev.

[33] Hines EM, Dowling S, Hegerty F, Pelecanos A, Tetsworth K (2021). Bacterial infection of fasciotomy wounds following decompression for acute compartment syndrome. Injury.

[34] Rickard J, Beilman G, Forrester J, Sawyer R, Stephen A, Weiser TG, Valenzuela J (2020). Surgical infections in low- and middle-income countries: a global assessment of the burden and management needs. Surg Infect (Larchmt).

